# Perceptions and processes influencing the transition of medical students from pre-clinical to clinical training

**DOI:** 10.1186/s12909-020-02186-2

**Published:** 2020-08-24

**Authors:** Bunmi S. Malau-Aduli, Poornima Roche, Mary Adu, Karina Jones, Faith Alele, Aaron Drovandi

**Affiliations:** College of Medicine and Dentistry, James Cook University, Townsville, 4811 Australia

**Keywords:** Transition framework; education, Students, Medical education, Clinical transition

## Abstract

**Background:**

The transition from pre-clinical to clinical medical training is often characterised by several challenges which may have different impacts on students’ well-being and learning experiences. To ensure smooth transition, it’s important to understand how these students navigate through the challenging processes.

**Methods:**

This study employed a mixed-methods design using a survey, focus groups and interviews among medical students who had entered their first clinical year of study (Year 4). Using a 5-point Likert scale, survey participants rated items which related to their transition experience in the areas of professional socialisation; workload; patient contact; knowledge and skills; and learning and education. The qualitative questions explored challenges in transition, coping strategies and recommendations to foster smooth transitioning. The survey data was analysed using descriptive and inferential statistics while thematic analysis was used to establish emerging themes from the qualitative data. The Westerman Transition Framework was utilised in the triangulation of study findings.

**Results:**

A total of 141 students participated in the survey while 12 students participated in the focus group discussions and interviews. The quantitative part of the study showed that the students were anxious about the process and considered the workload to be heavy while also identifying gaps in their knowledge. Similarly, the qualitative findings revealed that workload and professional socialisation were identified as disruptive novel elements and the students also reported feelings of inadequacy and incompetence due to perceived knowledge gaps. These shortcomings and challenges were tackled by seeking support from peers and senior medical students as a way of coping with the anxiety and stress. As the students progressed, they admitted and accepted that the transition was a gradual process and an essential learning curve.

**Conclusion:**

The process of transitioning from preclinical to clinical years is considered stressful and abrupt with the introduction of disruptive novel elements that create feelings of incompetence and unpreparedness in students. Educators need to consider developing social and developmental strategies that emphasise nurturing and empowering clinical learning environments and facilitate reflective and transformative life-long learning opportunities for students.

## Background

Transition from pre-clinical to clinical training has been reported as an exciting phase of learning for students due to changes in context and responsibilities [1–3]. However, this transition phase has also been suggested to be a source of stress and anxiety among medical students and this may be related to the perceptions of preparedness for clinical training [4]. Given that stress can be inhibitive to learning [5], educators are advised to better educate and prepare students for the changes that occur during the process in order to ease the transition [6].

Key areas where students encounter changes during this transition include professional socialisation; workload; patient contact; knowledge and skills; and learning and education [7]. Professional socialisation in medical practice involves the internalisation of a set of values and the culture of the profession [8], and this becomes imperative when students enter the clinical learning phase. Students have to develop a sense of belonging as members of the profession, integrate the values of their profession, and exhibit these values through their actions and behaviours [9]. Students have to navigate the hidden curriculum which is embedded as part of the medical culture and often contradicts lessons learnt and taught in the pre-clinical years [10]. Students experience the need to adjust to a new environment, and are often uncertain about what is expected of them or their role [11]. In addition, students experience an increased workload characterised by long working hours [12] and the need to adjust to the work routine [13] in the medical profession. Difficulty with time management and insufficient study time have also been reported as significant issues during clinical training [13]. Students’ early attainment of these professional expectations is essential to function well in their duties, thus making professional socialisation the foundation of effective practice [14].

Furthermore, perceived unpreparedness in knowledge and skills application to real patient problems usually leads to perceived inadequacy among students [7]. This is marked by feelings of disparity between the attained basic science knowledge of pathophysiology and diagnostic reasoning in clinical settings [1, 12]. In addition, students need to adopt different learning strategies that foster active learning [15, 16] coupled with the need to develop analytical reasoning skills [12]. They perceive the period of transitioning as that requiring progression from a diadatic teacher-centered model of learning to a problem-based student-centered model [15].

### Framework for transitioning

From a transitional psychological perspective, transitioning is described as a complex process of developing new behavioural responses to cope with some form of discontinuity in an individual’s life space [17]. When transitioning into the clinical phase, students encounter learning challenges which require them to develop task mastery, acculturation, role clarity and social integration [18]. The Westerman transition framework distinguishes between disruptive elements in medical transition, coping strategies, and personal development and outcomes in the transition process [19]. This transition framework was utilised in the present study as it describes the important concepts embedded in the process of transitioning in medical training. Disruptive elements refer to tasks that are new experiences required of students and encountered as a result of the new environment (transition from pre-clinical to clinical studies). Encountering new tasks usually instigates the development of coping strategies to adapt to the new environment and/or ease the intense transition [19]. Research shows that seeking opinions from seniors, learning among peers and resilience are correlated with good coping and positive adjustment among students [20, 21]. Coping strategies in clinical transitioning are necessary for successful studies and can be facilitated by factors such as expectations and personal resilience. Furthermore, as described by Westerman [19], personal development and outcomes reflect intrinsic potentials or qualities attained through development of coping strategies, which eventually contribute to achieving the required task. In addition, there are factors in the context of an individual’s personal life and clinical setting inherent to these elements of transition. A recent scoping review on the transition to undergraduate clinical training emphasised the need for research studies that explore the social and developmental perspectives of this phase of transitioning [19]. This approach focuses on building inter-personal relationships and empowerment of the student body to achieve smooth transition.

Therefore, this study aimed to examine the perception of students regarding transitioning from pre-clinical to clinical studies in the James Cook University (JCU) undergraduate medical training program. The study also utilised Westerman’s conceptual framework to explore the students’ social and developmental perspectives on this phase of transitioning. The study outcomes could provide useful insights to medical educators and faculty on evidence-based educational strategies to foster smooth transition.

## Methods

This study employed an explanatory mixed-methods approach which included the collection and analysis of both quantitative (through surveys) and qualitative data (through focus groups and interviews).

### Institutional context

James Cook University’s College of Medicine and Dentistry offers a 6-year undergraduate training program with a medical curriculum which is delivered with clinical exposure from Year 1. The first 3 years of the program are pre-clinical with introduction to the foundations of medicine and clinical experiences through simulated clinical skills sessions. The final 3 years are clinical years and involve rural and remote hospital-based placements/rotations, with the sixth year designated as the pre-intern year. The fourth year at JCU is the transitional link between preclinical teaching in years 1 to 3, to the clinical years 5 and 6. It introduces clinical pathology, clinical skills, clinical reasoning and prepares students for the clinical rotations in year 5. This is achieved in a clinical context through a series of integrated clinical pathological cases, simulated clinical skills workshops and ward-based learning.

### Participants and procedure

In September 2019, all Year 4 students (199 students) enrolled in the medical program were invited to participate in this study. To encourage participation, the research project was advertised in class 1 week prior to administering the survey. Additionally, as an incentive, participants were given the opportunity to enter into a draw to win one of three available $50 gift cards. Students were assured of no adverse academic repercussions for non-participation, with staff not associated with the teaching of medical students responsible for data collection and interacting with students. Informed consent was obtained from all participants.

### Data collection

#### Survey

A validated survey tool developed by Prince et al., [7] was distributed to students in September 2019. The tool explored student experiences in relation to five transition-related issues/ domains: professional socialisation; workload; patient contact; knowledge, knowledge application and skills, and learning and education. The 78 survey items consisted of statements rated on a 5-point Likert scale (1 = strongly disagree, 5 = strongly agree), which were followed by three open-ended questions related to perceptions on: 1) challenges experienced in the process of transition, 2) coping strategies for adaptation and 3) suggestions on supportive measures which could foster smooth transition. In this study, all participants were invited to attend either a focus group discussion or interview (depending on their preference) to allow for further exploration of their experiences in transitioning from pre-clinical to clinical training.

#### Focus group discussions and interviews

Focus Group Discussions (FGD) and individual interviews were conducted in October 2019 with the aim of gaining in-depth understanding that cannot be gained from a survey alone. The study was conducted towards the end of the clinical rotations and academic year, about 6 weeks before final exams. This timing allowed for student reflection on their experience through the year, thereby increasing their ability to provide information on their perception and experiences during their transition. The interviews and FGD took place in informal classroom settings, were audio recorded and lasted about 30–60 min each, with honesty and confidentiality emphasised. The discussions were based on semi-structured open-ended questions which were generated based on survey findings as well as existing literature. Questions asked during the FGD and interviews explored participants’ experiences of moving from theoretical to clinical training, building clinical skills, most difficult adjustment situation/problem encountered, strategies for coping with stress and suggestions to aid better adjustment to clinical studies.

### Data analysis

For the quantitative data, descriptive statistics including frequency, mean and standard deviations were calculated. Additionally, a Man-Whitney U test was conducted to investigate the relationship between demographic variables (age and gender) and the five [5] transition-related domains. For the qualitative data analysis, the FGD and interviews were audio taped, transcribed and coded by three of the researchers (MA, FA and BMA), with discrepancies resolved through a consensus meeting. Collated data were coded using a line by line open coding process. Emerging themes were identified using a constant comparison process, as advocated by Corbin and Strauss [22]. Subsequently, themes were analysed using Westerman’s conceptual transition framework [19]. The identified themes are presented using illustrative quotes which are affixed with participant gender, an assigned number and the type of data collection method (for instance Male #6, FGD). Triangulation of data was used to increase the rigour, validity and trustworthiness of this research. This process included utilisation of the quantitative findings to inform the qualitative phase of the study, with a subsequent integration of results from both phases to provide further explanations / interpretation of the overall study findings.

## Results

### Demographics

The response rate for the survey was 71% (*n* = 141), with a mean age of 22.19 ± 2.16 (19–39 years); most of the respondents were females (61%), had no prior health experience (92%) and were domestic students (89%). Full details of survey participants’ demographics are presented in Table 1. Nine students (5 males and 4 females), participated in the FGD, while three students (2 males and 1 female) were interviewed.

**Table 1:** Demographics of Participants (*N* = 141)

	Frequency	Percentage
**Gender**		
Male	55	39
Female	86	61
**Enrolment status**		
Domestic	125	88.7
International	16	11.3
**Age (Years)**		
19–23	107	82.3
24 and above	23	16.3
Missing	11	7.8
**Prior Health Experience**		
Yes	12	8.5
No	129	91.5
**Health Professionals in Family**		
Yes	67	47.5
No	74	52.5
**First in the family to attend**		
Yes	17	12.1
No	122	86.5
Missing	2	1.4
**Rurality**		
Major city	54	38.3
Regional centre	42	29.8
Rural town	37	26.2
Remote community	8	5.7

### Survey results

The mean scores for all Likert-scale statements are summarised in Table 2 and range from 3.1 to 3.9 out of 5. The mean scores for the five assessed domains were 3.4 for transition and professional socialisation, 3.3 for workload, 3.1 for patient contact, 3.6 for knowledge, knowledge application and skills, and 3.9 for learning and education. Overall, in all the domains, there were no significant differences in perceptions between the younger and mature aged students. However, gender difference was noted in only one of the domains - females reported a higher rating for transition and professional socialisation compared to their male counterparts (Median: 3.47 vs 3.37 Man Whitney U 1584.5000, *P* = 0.002).

**Table 2:** Mean scores for the Likert-scale statements by domain, arranged from highest to lowest mean score

Statement	Mean	SD	A (%)	N (%)	D (%)
**Domain 1: Transition and Professional Socialization**	**3.4**				
Collaboration with other medical students on clinical placement/rotation was easy	4.0	0.9	81	13	6
A general introduction should be provided to all new medical students on clinical placement/rotation	4.0	0.8	78	19	4
A good introduction would make the transition easier	3.9	0.9	74	20	6
I enjoyed the first few weeks	3.8	0.9	77	11	11
My first clinical placement/rotation proved to be better than I expected	3.8	0.9	73	17	10
The clinical staff were easy to work with	3.8	0.8	71	22	7
The introduction into the clinical placement/rotation was satisfactory	3.5	0.8	59	29	12
I felt ready to begin clinical training	3.5	0.9	55	33	13
My uncertainty lasted only a few days	3.3	1.2	48	29	23
The transition from pre-clinical to clinical training went smoothly	3.2	1.0	45	29	26
I needed time to adjust to the new environment	3.8	1.0	70	17	13
I was nervous at the beginning of the clinical placement/rotation	3.7	1.0	68	17	15
I felt well prepared for clinical training	3.2	0.9	44	35	21
I experienced an abrupt transition from pre-clinical to clinical training	3.2	1.1	44	26	31
I experienced a great deal of stress	3.2	1.2	42	26	33
I was very uncertain at the beginning of the clinical placements	3.1	1.1	42	24	34
This was the first time I experienced what it is like to work as a doctor	2.9	1.2	36	21	43
The first few weeks on clinical placement/rotation were difficult for me	2.8	1.0	29	26	45
I have considered quitting medical school	2.6	1.4	31	14	56
**Domain 2: Workload**	**3.3**				
There is a huge difference between my workload before and after the transition to clinical training	3.6	1.0	61	21	19
The workload of medical students on clinical placement/rotation is heavy	3.5	0.9	59	24	18
So far clinical placement/rotations have been tiring	3.5	0.9	58	26	16
I had difficulty getting used to the work routine	3.1	1.1	39	24	37
As a medical student on clinical placement/rotation I have to work very long hours	3.0	1.0	33	32	35
As a medical student on clinical placement/rotation I have enough time to study	2.9	1.0	36	28	36
**Domain 3: Patient Contact**	**3.1**				
The knowledge that I acquire from contact with real patients is easier to retain	4.3	0.8	89	8	3
Contact with real patients is easy for me	3.9	0.8	76	18	6
Contact with real patients stimulates me to study	3.9	0.9	74	20	6
I would have liked real patient contact earlier in the curriculum	3.8	1.0	64	25	11
I think patients feel uncomfortable when they are examined by a student	2.6	1.0	22	28	50
My first contact with real patients was during the clinical placement/rotations	2.2	1.2	22	4	74
I feel uncomfortable when I examine a patient	2.2	1.0	14	17	69
I am afraid to start a conversation with a patient	1.9	0.9	7	13	80
**Domain 4: Knowledge, knowledge application, and skills**	**3.6**				
I am able to do a *full* physical examination	4.2	0.6	93	7	1
I am able to take a history	4.1	0.7	88	10	2
I am able to do a physical examination	4.0	0.6	86	11	3
In clinical practice other aspects of knowledge are important than during pre-clinical training	4.0	0.7	76	23	1
I am able to apply my knowledge in practice	3.8	0.6	78	19	3
The knowledge required in clinical practice is different from my theoretical knowledge	3.7	0.9	68	22	10
I felt well prepared to perform medical technical skills	3.7	1.0	67	18	15
There are gaps in my knowledge	4.2	0.8	87	9	4
The knowledge I acquired during the pre-clinical phase is relevant for the clinical phase	3.7	0.9	66	24	10
I have sufficient clinical science knowledge	3.7	0.9	50	35	15
I feel confident about the findings from history and physical examination	3.6	0.7	58	35	7
I have sufficient behavioural science knowledge	3.4	0.9	53	27	19
When I do a history and physical examination, the findings are checked by clinical staff	3.3	1.2	50	23	28
I have sufficient basic science knowledge	3.3	0.8	46	32	22
I have the appropriate knowledge readily available	3.3	0.8	46	37	17
I felt well prepared for clinical skill performance	3.2	0.8	40	43	17
I felt well prepared with respect to communication skills	3.2	0.9	40	36	24
I was sufficiently prepared for the clinical placement/rotations as regards theoretical knowledge	3.1	0.9	36	35	29
The level of my knowledge is sufficient	3.1	0.9	36	41	24
I have difficulty recognising pathological symptoms	2.6	0.9	19	29	53
**Domain 5: Learning and Education**	**3.9**				
Junior medical doctors are good teachers	4.4	0.7	92	7	1
Senior medical students are good teachers	4.4	0.6	92	8	0
I learned a lot from real patient tutorials	4.3	0.6	94	5	1
Real patient tutorials were good preparation for the clinical placement/rotation	4.3	0.6	92	7	1
In clinical practice I study in a different way	4.2	0.7	91	8	2
You can learn a lot from bedside teaching	4.2	0.8	83	12	4
The knowledge I acquire in clinical practice is easier to remember	4.1	0.7	88	9	3
Case based learning provided good preparation for clinical practice	4.1	0.7	86	12	2
I am able to study independently	4.1	0.9	84	9	7
I learned a lot from the tutorial meetings in which the simulated patient contacts were discussed	4.0	0.7	80	18	2
Volunteer simulated patient contacts were good preparation for contact with real patient	4.0	0.8	79	16	5
I need to study because I have forgotten a good deal of my theoretical knowledge	4.0	0.9	75	19	7
I learned a lot from simulated patient contacts	3.9	0.8	75	19	6
Senior medical doctors are good teachers	3.9	0.8	75	18	7
I study in a different way in clinical practice than during my first years in medical school	3.8	1.0	73	12	16
You can learn a lot from the handover of patients	3.8	0.9	68	21	11
I study more intensively than before the clinical placement/rotations	3.7	1.0	64	18	18
I study to learn the things that I want to know	3.7	0.9	63	26	11
It is easy for me to obtain experiences from which I can learn	3.6	1.0	61	27	12
What I study is influenced by the assessment program	3.6	1.0	64	18	18
I study primarily for tests and examinations	3.4	1.1	49	27	25
The first years in medical school were relevant for clinical practice	3.3	1.0	51	29	20
My learning is driven by questions from clinical staff	3.3	1.0	49	30	21
What I study depends on the problems I have encountered that day	3.3	0.9	48	28	25
I am able to judge my own progress	3.2	1.0	44	30	25
**Additional Items**					
My first semester of clinical work reassured me that I had made the right choice of vocation/career	4.0	0.85	75	21	4
I enjoy studying in a group setting	3.6	0.98	61	27	12

*A* Agree, *N* Neutral, *D* Disagree

#### Transition and professional socialisation

As shown in Table 2, although a larger percentage (68%) of the students were nervous at the start of clinical placement, more than half (55%) felt mentally ready to begin the placement. Almost an equal proportion of them felt well prepared for clinical training (44%) and were able to transition from pre-clinical to clinical training smoothly (45%). Even though many enjoyed the first few weeks of placement (77%), they also needed time to adjust to the new environment (70%). Nearly half of the participants experienced an abrupt transition from pre-clinical to clinical training (44%) and indicated being stressed (42%). Many were able to collaborate easily with their peers on placement (81%) and other clinical staff (71%). Most students were satisfied with the introductory/ orientation session given by the faculty (74%), acknowledged the importance of a good orientation for easier transitioning, and supported the provision of a general introduction for all new students on clinical placement (78%). As shown in Table 3, the significant gender differences observed in the transition and professional socialisation domain were in relation to anxiety at the beginning of the clinical placement with more females reporting that they felt nervous (*P* < 0.001) and uncertain at the beginning of the clinical placement (*P* = 0.010). Similarly, females felt the transition from pre-clinical to clinical training was abrupt (*P* = 0.033) and they needed more time to adjust to the new environment (*P* = 0.002). In addition, females reported that they experienced a great deal of stress compared to their male counterparts (*P* = 0.021). However, the clinical placement proved to be better than expected for females compared to males (*P* = 0.049).

**Table 3:** Significant Likert scale statements in Transition and Professional Socialisation in relation to gender

Item	Female		Male		Mann-Whiney U	***P*** value
	Mean	Median	Mean	Median		
I was nervous at the beginning of the clinical placement/rotation	3.96	4.00	3.28	3.00	1430.50	< 0.001
I was very uncertain at the beginning of the clinical placements	3.31	4.00	2.79	3.00	1718.00	0.010
I needed time to adjust to the new environment	4.00	4.00	3.45	4.00	1646.50	0.002
I experienced an abrupt transition from pre-clinical to clinical training	3.32	4.00	2.94	3.00	1843.00	0.033
I experienced a great deal of stress	3.36	3.00	2.85	3.00	1798.50	0.021
My first clinical placement/rotation proved to be better than I expected	3.96	4.00	3.66	4.00	1899.50	0.049

#### Workload

Almost a third (33%) of the students felt they worked very long hours on placement while over half of them perceived the workload as heavy (59%) and found the placement tiring (58%). Nearly 40% had difficulty adjusting to the clinical work routine and 61% perceived that a huge difference exists between their pre-clinical and clinical workload (Table 2).

#### Patient contact

For most participants, contact with real patients was easy (76%) and knowledge acquired from this contact was easier to retain (89%). However, many (64%) would have appreciated an earlier patient contact opportunity in the curriculum (Table 2).

#### Knowledge, application of knowledge and skills

Majority of the students (87%) perceived gaps in their overall knowledge, where only about half of them felt they had sufficient knowledge in basic science (46%), clinical science (50%) and behavioral science (53%). Many participants felt the knowledge acquired during pre-clinical phase is relevant for the clinical phase (66%) and were able to apply the knowledge to practice (78%). Students were of the opinion that clinical practice required essential knowledge that was not emphasised during pre-clinical training (76%). Nonetheless, they felt the knowledge acquired during pre-clinical years, in relation to physical examination (93%) and history taking (88%) procedures were highly applicable in the clinical years. Majority of the students felt well prepared to perform medical technical skills (67%), but a lower percentage felt prepared for core clinical (43%) and communication (40%) skills and a few still had difficulty recognising pathological symptoms (19%).

#### Learning and education

Students felt what they learnt in clinical placement was easy to retain (61%), their learning was guided by their area of interest (62%) and the assessment program (64%). Most of the students attested to studying in a different way during the clinical practice (91%) and more intensively (64%). Three-quarters (75%) of the cohort felt they needed to revisit the basic sciences because they had forgotten most of their theoretical knowledge.

Only half (51%) of the students felt that the preclinical training was relevant to practice but more participants (86%) agreed that case-based learning gave them a good preparation for practice. Majority of the participants reported that simulated patient contact was a good preparation for learning (79%) and they had learnt a lot through this (75%) and through tutorials (80%). More participants were attuned to learning from bedside teaching (83%) than during handover of patients (68%). Interestingly, many participants (92%) perceived junior academic staff and senior medical students as good teachers. The first semester of clinical placement reaffirmed to many students (75%) that they had made a right career choice.

### Qualitative results

The emerging themes from the qualitative data are presented in relation to the transition context and their accompanying tasks as encountered by students. Given that the original framework explored the transition of medical ‘residents’ to ‘attendings’, some subthemes were not applicable to our study population. The themes that emerged were adapted to reflect the transition experiences of medical students from pre-clinical to clinical learning within our context (Table 4). The three overarching elements of the framework (disruptive novel elements, perceptions and coping strategies, and personal development and outcomes) were retained, and themes are presented within these elements.

**Table 4:** Overview of the challenges and adaptations identified by the participants

Domain	Challenge	New Task/ Adaptation
Disruptive novel elements	Altered learning method	Self-directed learning, Application of knowledge to clinical context
	Medical work culture	Increased workload, Professional relationships, Changed socialisation context
Perceptions and coping strategies	Perceived inadequate preparation	Learning on the run, Seeking support/ guidance
Personal development and outcomes	Accepting the new reality: gradual process	Identifying alternative learning methods, Reduced feelings of incompetence

### Disruptive novel elements

#### Altered learning methods

One of the new tasks encountered in clinical training was self-directed learning. Students noted this was particularly challenging due to their inability to deduce the specific topics to focus on when studying. The participants also reported the lack of frequent structured lectures in comparison to what was obtainable in pre-clinical years.

*“Self-directed nature. Sometimes it can be hard to determine the depth of knowledge we require.”* [OER]

As indicated by the students, the major challenge with self-directed learning was the inability to check the progress of learning.

“*We don’t actually have any lecture on the full knowledge we are supposed to be gaining. So, I think it’s easier to fall through and fall behind because you are not having those land marks of every week, like I’ve got GLS and then in 6-10 weeks, you are like…I haven’t been reading anything…You have [been] doing the completely wrong thing because you don’t have those sort of goal post or landmarks to tick off.*” [Male #7, FGD].

Application of pre-clinical knowledge to clinical setting was also a challenging task for the students. Students perceived that difficulty in knowledge application stemmed from the self-directed nature of this phase of transition because they did not know the breadth and depth of learning required.

“*Well, it's just difficult, like, what would I say? Um, so it's just challenging for me because we've learned all the physiology in the first three years. Then, this year is sort of where you really see all the pathology, and then just sort of link in and understand what's going on in your patients.”* [Male #1, INT]

“*Everyone transitioning is definitely going I don’t know what I need to know. That's the biggest thing. Although there are resources given and we do have a list of diseases that we need to know, but it's the extent of how much we need to answer and like gauging how much we need to know about, say, a particular renal disease or something like that. Like how much depth do we need to go into?”* [Male #3 FGD].

The participants reiterated the need to revisit basic science subjects like anatomy. There were concerns that some clinical rotations like radiology required a sound knowledge of anatomy which some of the students had already forgotten.

*“Anatomy is the one that is lacking the most in teaching. It's almost non-existent in fourth and fifth year.”* [Male #3, FGD]

Interestingly, there were gender differences in relation to perceptions of the transition process. Males expressed more excited opinions about the process and its impact on their learning while females were more anxious. This confirms the gender differences reported in the quantitative part of the study. “*So, I think for me, the first semester or the first half of the year was easy. I was transitioning and more so just enjoying the clinical environments. Like whoa, this is really cool. Like I get to hang out with clinicians and see all these cool things, rather than actually studying. So, I wouldn’t say it was stressful for me*” (Male #5 FGD)

“*I think that's a bit much because last year, you got a lecture, you got a GLS, a three hour session, [and now] it’s like just dropping us into the middle of nowhere … .good luck with the CLIs, good luck with your CPC. I just felt like I needed more teaching, more one-on-one, like the CLIX sessions*” (Female #2 FGD).

#### Medical work culture

Participants identified a change in task characterised by increased work load, where managing the time between work and clinic duties was demanding. This was especially noted by students who also balance studies with external work commitments.

“*There are realities of paying rent and as students we’re really contending with that and unfortunately we all don’t have scholarships or our way paid. Some people have to work and it is very frustrating*” [Female #2, FGD].

The workload involved in clinical training in medicine which requires time commitment and total dedication was termed as unrealistic:

*“We are told our time at Med school is from 8:00am to 6:00pm because we’ve signed for this… I think this is a sheltered view to think that students don’t have other responsibilities.”* [Female #1, INT]

*“Somebody said at the lecture if you work, that is now your second priority to medicine.”* [Female #3, FGD]

Interpersonal professional relationships in the clinic environment was a novel experience for students. This task demands confidence that some participants sometimes found themselves lacking. As one participant observed.

“*The most difficult thing for me was approaching the doctors and introducing myself to everyone. So, just building the confidence to go to people especially when they are busy and you have to interrupt what they are doing and introduce yourself”* [Male 2, INT].

The new learning environment also meant separation from their circle of friends as they may not be on the same clinical rotation, thereby changing their socialisation context.

*“So yeah, that was a big thing, so I don’t have the strongest friendship group as I did in the first three years, now. Now I kind of stick with my rotation mainly, because we're all together all the time anyway”* [Female #1, INT]

### Perceptions and coping strategies

The disruptive novel elements encountered during the transition were addressed through a range of coping strategies. Participants perceived the period of change surrounding the transition from pre-clinical to clinical years as that which signifies learning on the run in the workplace, typical of medical studies.

“*I think the explanation as it is in medicine is, you learn as you go because it is so hard to teach all of that (clinical skills application).*” [Female #2, FGD]

Although participants highlighted that they enjoyed the clinical training, feelings of inadequate preparation were evoked by their perceived inadequate knowledge in some of the core subjects which are relevant to clinical work. Participants believed transitioning would have been easier if there had been more detailed information on expectations in training and consolidation of ideas across core subject areas in pre-clinical years which detailed the applicability to clinical work. Participants used various coping strategies to tackle these new challenges. Most frequently mentioned was the need to seek support from their seniors:

“*Working with senior students and interns have helped me prepare for the clinical years. They have provided useful advice and suggestions for resources which have been helpful.*” [OER]

This was in addition to being proactive in seeking advice and guidance from their peers.

“*I just ended up talking to as many people as I can and find out what people are doing, what they find works. I think a lot of people did that, so we kind of basically tried to suss it out together”*. [Male #6, FGD],

*“There’s lot of knowing what you need to know by working in study groups as well as practising your clinical skills with each other, which has really been handy this year.*” [Female #3, FGD].

Feeling supported by tutors was of utmost importance in attaining knowledge and applicable clinical skills. The semi-structured support provided by tutors fostered knowledge acquisition which was perceived to be beneficial for both skills development and confidence building.

“*Clinical skills has been really valuable this year because we’ve had good tutors and I like the structure where we have three weeks on a different system so we start with the tutors, perform it in front of everyone. Then we have a quick go at it ourselves, and the next week we have another go at it on patients*.” [Male #5, FGD]

### Personal development and outcomes

Participants realised that successful transition from pre-clinical to clinical years is a gradual process which requires adaptation.

*“It’s just something you have to adapt to, because it’s not what we’re used to with the first three years, not that the structure is wrong but its’ just different so we have to adapt to it.”* [Male #1, INT]

Initial challenges including feelings of inadequacy and incompetence subsided over time and the students were able to adopt suitable learning methods/ styles that helped them develop the knowledge and skills required for the job.

*“I guess just slowly building the confidence to do it and, yeah, because it really is the right thing to do.”* [Female #1, INT]

*“So yeah, definitely just studying in groups basically has probably been the best way to do it, especially like if it's productive and you're just really, yeah, I suppose, just working as much as possible and everything like that, and working out your learning…But that's a difficult thing to sort of figure out. Like I didn't figure it out until probably, what, a month ago or something.”* [Male #3, FGD]

## Discussion

Students’ self-perceived readiness to transition from the pre-clinical to the clinical phase of their learning was a dominant outcome in this study, with both enabling factors and barriers influencing the transition positively or negatively respectively. The transition is often a stressful period, with students stating that they needed more time to adjust to the clinical environment despite enjoying the first week and feeling mentally ready. This associated stress and anxiety is well documented in the literature [1, 7, 23]. The findings of this current study buttresses previous findings where stress and anxiety experienced can be attributed to perceived knowledge gaps between pre-clinical and clinical training, pressures of medical work culture/ professional socialisation and from a developmental perspective, the need to develop self-directed learning skills [1, 2, 7, 12].

Integration of the findings from the two phases of this study revealed that female students considered the transition to be abrupt, needed more time to adjust, were nervous and uncertain at the start and reported a higher perceived level of stress compared to males. The emphasis on the abruptness of the process and associated stress was due to the limited guidance during the process, corroborating the results of previous studies that reported increased perceived stress levels among females indicating that the transition process may be more challenging for them compared to their male counterparts [24, 25].

From an educational perspective, students identified perceived shortcomings in basic science knowledge as well as perceived difficulty in putting theory to practice. The students felt that they had either forgotten foundational knowledge (such as anatomy), had gaps in their knowledge, or struggled to integrate materials that were inter-related. Previous research by Prince et al. [7], reported similar findings. Educators need to adopt teaching styles that move students away from traditional lectures earlier in the curriculum, with the incorporation of more patient-based clinical learning which the students found useful to aid retention of knowledge and skills acquisition [26]. This would improve their communication and clinical skills before exposure to clinical placements and assist them in translating pre-clinical knowledge into clinical settings outside of the high-pressure clinical placements [26]. Providing early patient contact through placements [27] and longitudinal integrated clerkships [28] can also assist in preparing students for transition to the clinical environment.

The study participants reported some difficulty in adapting to the work culture in the hospital setting and establishing working relationships with senior health professionals. This is also consistent with findings in the literature where students felt intimidated in the hospital environment [11, 23] and struggled to understand the prevailing medical culture [29]. Although there were challenges associated with establishing interpersonal relationships; other aspects of professional socialisation such as developing a professional persona or role modelling were not identified as stressors in this study [1]. Similarly, the hidden curriculum which refers to unintended learning experiences (including professional norms, beliefs, and values) was not identified as a challenge during this transition phase [30]. Given that this was the introductory year to clinical training for the participants and they are still new to the system, it is possible that they may not have a full understanding of the medical culture and would require more time to identify unintended learning experiences in this context. That may in part explain why the hidden curriculum was not identified as a stressor. Medical educators could prepare students for professional socialisation by including more comprehensive and early gradual orientations to the clinical environment. This allows the students to gradually comprehend and adopt the values, attitudes and normative behaviour of the medical profession [31]. In addition, the difficulty associated with balancing work responsibilities and academic needs among the students has been documented in previous research [32]. Medical educators need to discuss and address these issues with students before they move into the clinical training years [32]. Furthermore, encouraging and guiding students to set realistic and attainable learning objectives in relation to clinical training may make it easier for them to navigate work-life balance [26].

Although the perceived stressors were novel for the students and were perceived to disrupt the known way of learning; they may be considered as a normal aspect of entering into a new work environment [12]. This transition was regarded by the participants as a longitudinal process of change which created discontinuity in their educational or training process, forcing them to develop new behavioral responses to cope with the changes and challenges encountered [19]. The students stated that utilising available support systems from senior students, tutors, and recent graduates was an essential coping strategy to overcome the uncertainties and enhance application of theoretical knowledge to clinical context. This emphasises the importance of guided and near-peer support programs during the transition from preclinical to clinical training. Educators should consider promoting formal near-peer support systems, which facilitate interactions with senior students and academics, throughout the medical degree to make the transition process easier for students [1].

Despite the perceived stress and anxiety associated with the transition process, the participants were cognisant that it was a gradual adaptation process which requires time and patience. As an ever-changing longitudinal process, transition requires adaptability and resilience to cope with the expectations of the new role [19]. Evidence suggests that resilience is a measure of the ability to cope with stress and is a way to mitigate the negative effects of stressors and help students succeed after difficult experiences [33]. It may be important for resilience and mental wellbeing training programs to be incorporated into medical education given that medical students may encounter new and salient stressors during their training [33, 34]. In addition, medical students transitioning from preclinical to clinical training need to develop lifelong self-directed learning skills for continuing professional development in clinical training [35]. Additionally, it is important for medical educators to ensure that disruptive elements or negative stressors for the students are minimised during the transition phase to reduce stress and anxiety while also empowering students with strategies that promote personal and professional development.

### Implications for practice

The findings of this study provide evidence for explicit communication of performance expectations, especially in relation to professional socialisation and application of basic science knowledge in clinical contexts. The uncertainty around application of knowledge highlights the importance of early and gradual integration of foundational and clinical knowledge and diagnostic reasoning to ease the transition process. Medical educators need to consider the development and application of instructional models that alternate skills training with some clinical exposure in the pre-clinical years. This approach will aid gradual immersion of medical students into the clinical setting with mastery of clinical concepts within simulated safe learning environments to foster confidence for the application of learned knowledge and skills to the clinical context [36]. To counter perceived barriers (such as nervousness and lack of confidence) in building team relationships while in the clinical environment, medical educators should consider developing mentoring and near-peer transition support programs that gradually introduce the students to clinical years [26]. The use of near-peers including recent graduates to provide teaching support to students transitioning into the clinical year would serve as a valuable tool given the recent experiences of such support groups and their ability to understand the struggles of the transitioning students [37]. The transition programs should have specific and measurable objectives that are relevant to students’ performance and participation in the clinical training [26]. This will aid the development of interpersonal and clinical skills, provide a better understanding and application of the foundation sciences, improve confidence and alleviate the feelings of inadequacy and anxiety [38]. It is also important for clinicians involved in the education of the students to provide learning environments that foster collegiality to support students’ learning and reduce stress. Additionally, pedagogical training of clinical tutors and educators should be emphasised to ensure gold-standard teaching practices and alignment between teacher-student expectations.

### Strengths and limitations

The major strengths of this study are the utilisation of mixed methodology which employed quantitative and qualitative approaches and a concept-driven framework analysis to identify the perceptions and processes involved in transitioning from preclinical to clinical years among medical students. Triangulation of the quantitative and qualitative findings offered a comparative analysis that revealed interpretations of convergent findings and ensured data trustworthiness. However, generalisations of the study findings may be limited by the fact that this study reports findings from a single institution and the smaller number of participants in the qualitative phase of the study. Additionally, it is possible that the volunteer participants may be outliers who have high academic expectations and anxieties or those who have had negative experiences and wanted to express their views.

Nonetheless, given that there was a good representation of the different student groups in both phases of the study and data saturation was achieved, the lessons learnt may be germane to a larger population and different settings. Furthermore, exploring students’ perceptions while actually experiencing the transition, allowed the researchers to accurately capture the experience and this approach yielded greater insights into the enablers, barriers and strategies that are effective for reducing the disruptive stress of transition. These strategies emphasise the creation of nurturing and empowering clinical learning environments that facilitate reflective and transformative life-long learning opportunities for students.

Finally, given that transition is a gradual process, it is imperative that longitudinal exploratory studies that make comparisons over time are conducted to further elucidate each stage of the transition process in greater detail. Furthermore, future studies should consider investigating the perceptions of the transition process among learners using case/ problem-based versus those from traditional-based curriculum. Identifying and comparing the perceptions of transition between the two groups will provide useful and valuable information that will aid curriculum development and reform.

## Conclusion

Transition into clinical training is an evolving process that requires time and resilience to adjust to the new role. Disruptive novel elements such as self-directed learning on the run, medical work culture and lack of confidence to engage in interpersonal work relationships can hamper professional socialisation and make the transition process unpleasant for students. Therefore, to ensure smooth transitioning, it is important for educators to develop pedagogical evidence-based strategies that minimise students’ anxiety and stress, optimise their learning opportunities and gradually immerse them into clinical training.

## Data Availability

All data generated or analysed during this study are included in this published article.

## Abbreviations

CPC: Clinical Pathological Case Studies
CLI: Clinical Intensive Rotations
CLIX: Clinical Investigations
FGD: Focus Group Discussion
GLS: Guided Learning Sessions
INT: Interview
JCU: James Cook University
OER: Open-ended Responses
